# Production and Characterization of a Polyclonal Antibody of Anti-rLipL21-IgG against *Leptospira* for Early Detection of Acute Leptospirosis

**DOI:** 10.1155/2014/592858

**Published:** 2014-04-22

**Authors:** Arivudainambi Seenichamy, Abdul Rani Bahaman, Abdul Rahim Mutalib, Siti Khairani-Bejo

**Affiliations:** Department of Veterinary Pathology and Microbiology, Faculty of Veterinary Medicine, Universiti Putra Malaysia, 43400 Serdang, Selangor, Malaysia

## Abstract

Leptospirosis is one of the zoonotic diseases in animals and humans throughout the world. LipL21 is one of the important surface-exposed lipoproteins in leptospires and the most effective cross protective immunogenic antigen. It is widely considered as a diagnostic marker for leptospirosis. In this study, we evaluated the serodiagnostic potential of LipL21 protein of *Leptospira interrogans* serovar Pomona. We have successfully amplified, cloned, and expressed LipL21 in *E. coli* and evaluated its specificity by immunoblotting. Purified recombinant LipL21 (rLipL21) was inoculated into rabbits for the production of polyclonal antibody. Characterization of the purified IgG antibody against rLipL21 was performed by cross reactivity assay. Only sera from leptospirosis patients and rabbit hyperimmune sera recognized rLipL21 while the nonleptospirosis control sera showed no reaction in immunoblotting. We confirmed that anti-rLipL21-IgG antibody cross reacted with and detected only pathogenic leptospiral species and it did not react with nonpathogenic leptospires and other bacterial species. Results observed showed that anti-rLipL21-IgG antibody has high specificity and sensitivity to leptospires. The findings indicated that the antibody could be used in a diagnostic assay for detection of leptospires or their proteins in the early phase of infection.

## 1. Introduction

Leptospirosis is a major public health concern and has now been identified as one of the emerging infectious diseases worldwide [1, 2]. It is highly prevalent in the Asia Pacific region [3]. In Malaysia, leptospiral infections seen in domestic animals were mainly due to serovars of the Sejroe and Pomona serogroups. The infections in humans were also due to* Leptospira interrogans *serovar Pomona [4]. It is considered that the majority of leptospirosis cases in humans were due to association with animals and disease-infected environment [5]. Leptospirosis displays a wide array of clinical presentations and it is difficult to distinguish from dengue, malaria, and influenza. It mimics many other diseases characterized by fever, headache, and myalgia [6]. After clearance of leptospires from blood and body fluids, they are known to persist for prolonged periods in immune privileged sites like renal tubules, brain, and eyes of carrier animals [7]. Leptospires include 268 serovars [8] and can be organised into 31 serogroups on the basis of their antigenic relatedness [5]. The antigenic structure of leptospires is complex, with structural heterogeneity in the carbohydrate component of the lipopolysaccharides (LPS) [7]. The outer membrane of leptospires is composed of immunogenic LPS and different types of outer membrane proteins (OMPs). Lipopolysaccharides play a key role in immunity to infection and are responsible for serovar variations [9]. Based on serological classification, the genus* Leptospira* was earlier classified into two species, the pathogenic* L. interrogans* and nonpathogenic* L. biflexa*.

The leptospiral membrane possesses at least three types of OMPs: lipoproteins, peripheral OMPs, and transmembrane proteins [10, 11]. Among them LipL21 was described as one of the most important outer membrane lipoproteins produced during leptospiral infection [12]. Detection and identification of conserved leptospiral lipoproteins among pathogenic leptospires were made through cross-protection assays against various serovars of leptospires. The exclusive presence of these proteins in the pathogenic leptospires indicated that they could be promising candidates for developing diagnostics [13]. Hence these lipoproteins have currently become a major focus of leptospirosis research [14].

In the present study, we expressed the recombinant protein LipL21 and evaluated its immunogenic potential with leptospirosis human sera and rabbit hyperimmune sera. We used purified, recombinant LipL21 to create polyclonal immunoglobulin G (IgG) against rLipL21. The results of the present study provide a possible new candidate for developing diagnostic kit for detecting leptospires and/or their leptospiral antigen during the early stages of infection.

## 2. Materials and Methods

### 2.1. Bacterial Strains and Growth Conditions

In this study, leptospiral reference strains were obtained from WHO Collaborating Centre Brisbane, Queensland, Australia, as shown in Table 1. Other bacterial strains used in the study include* E. coli, Pseudomonas aeruginosa*,* Staphylococcus aureus*,* Pasteurella multocida*,* Bacillus subtilis*,* Salmonella typhi*,* Klebsiella pneumoniae*,and* Proteus *spp. These were kindly provided by the bacteriology Lab, Faculty of Veterinary Medicine in University Putra Malaysia.* Escherichia coli* DH-5*α* (lab collection) and BL21 (DE3) (Novagen, Madison, WI) were used for cloning and expression to purify the recombinant protein. Leptospires were grown to mid-logarithmic phase for 7 days at 30°C in liquid Ellinghausen-McCullough-Johnson-Harris (EMJH) medium.  The* E. coli *strains were routinely grown in Luria-Bertani (LB) medium at 37°C, with appropriate selection pressure (ampicillin (50 *μ*g/mL), kanamycin (50 *μ*g/mL), and chloramphenicol (34 *μ*g/mL)).

### 2.2. Amplification and Cloning of LipL21

*Leptospira *spp. were grown in EMJH medium to mid-log phase for DNA extraction. Genomic DNA extracted from 1 × 10^8^ cells using the Promega Wizard genomic DNA purification kit (Promega, Madison, WI, USA). The new primers designed were based on comparison of 56601 sequences retrieved from GenBank (Gene ID: 1149354) of* L*.* interrogans* serovar Lai strain. The primers used for the amplification of* lipL21 *gene are FL21-5′GAG AAG *CATATG* ATC AAT AGA CTT ATA GC 3′ with restriction site* Nde*I and RL21-5′-CCC *GAATTC*  TTA TTG TTT GGA AAC CTC TTG-3′ with restriction site* Eco*RI. The resulting amplicon was digested with restriction enzymes* Nd*eI and* Eco*RI and inserted into pET-28(b) vector (Novagen). The recombinant plasmid construct was confirmed for accurate insertion by both restriction enzyme digestion and sequencing.

### 2.3. Expression of Recombinant LipL21 Fusion Protein

The pET28-LipL21 (pEL21) plasmid was transformed into* E. coli* BL21 (DE3) (Novagen) [15]. A single colony of* E. coli* BL21 (DE3) harbouring the pET28-LipL21 (pEL21) plasmid was inoculated into 10 mL LB media containing 50 *μ*g/mL kanamycin and incubated overnight at 37°C with shaking at 200 rpm. An aliquot of 100 *μ*L of the overnight cell culture was added to another tube of 10 mL LB medium (containing 50 *μ*g/mL kanamycin and Overnight Express Autoinduction System 1) (Novagen) and incubated at 20°C with shaking (200 rpm). The overnight induced culture was harvested aseptically by centrifugation at 12,000 g for 3 min.* E. coli* BL21 (DE3) cells harbouring the pEL21 vector were used as the uninduced or negative control. Bacterial pellets recovered after inductions were dissolved in appropriate volume of Laemmli buffer and proteins were resolved on a 12% SDS-PAGE. The expression of the recombinant LipL21 (rLipL21) was detected by Western blot using the His tag AP Western blot kit (Novagen). Protein concentrations were determined by using a bicinchoninic acid (BCA) protein assay kit (Bio-Rad).

### 2.4. Purification of rLipL21 Fusion Protein

For auto induction, 1 litre culture was incubated at 20°C for 18 h with orbital shaking (190 rpm), using the Overnight Express Auto Induction System 1 according to the specifications provided by the manufacturer (Novagen). After the induction period, the cells were collected by centrifugation for 10 min at 12,000 g at 4°C, resuspended in one-tenth of the culture volume of binding buffer (50 mM NaH_2_PO_4_, 300 mM NaCl, 10 mM imidazole, pH 8.0) and subjected to sonication (Branson ultrasonifier, USA) till complete cell lysis. The lysates of induced culture are cleared by centrifugation at 12,000 g for 30 min at 4°C and were applied on a His-Trap (Novagen) of Ni^2+^-nitrilo-triacetic acid (Ni-NTA) affinity column (2 mL). Ni-NTA column was preequilibrated with binding buffer (50 mM NaH_2_PO_4_, 300 mM NaCl, 10 mM imidazole, pH 8.0) and finally it was washed using wash buffer (50 mM NaH_2_PO_4_, 50 mM NaCl, 10 mM imidazole, pH 8.0) to remove the unbound proteins. Bound proteins were eluted with elution buffer (50 mM NaH_2_PO_4_, 300 mM NaCl, 250 mM imidazole, pH 6.0). The eluted recombinant LipL21 protein was dialysed and concentrated by Centriprep-30 (10 kDa cut off) (Millipore-Amicon, Beverly, MA). The protein concentration was determined by BCA method. The purity of LipL21 protein was analysed by 12% SDS-PAGE and visualized by staining with Coomasie brilliant blue [16].

### 2.5. Polyclonal Antibody Production

Polyclonal antibody production was carried out according to the method of Shang et al. [17]. Purified rLipL21 protein was loaded onto SDS-12% polyacrylamide gel and separated during electrophoresis. A rLipL21 containing band was excised from the gel and desiccated. The desiccated gel containing recombinant protein was ground to a powder, dissolved in 1 mL of water, and mixed with 1 mL of complete Freund's adjuvant (Merck, Whitehouse Station, NJ). New Zealand White rabbits (free of leptospiral antibodies) were immunized with the mixture of rLipL21 and complete adjuvant (subcutaneously and intramuscularly) on Day 1. Additional immunization with the same dosage of rLipL21 with incomplete Freund's adjuvant (Merck) was done on Day 14, Day 28. On Day 42 the rabbits were bled by heart puncture and the serum was tested to detect antibodies against LipL21. The LipL21-antiserum was stored in small aliquots at −20°C until use. Animals were housed in accordance with the ethical principles and experimental procedures with animals were approved by the Animal Care and Use Committee of the Faculty of Veterinary Medicine, University Putra Malaysia (AUP No: 09R57/Mac 09-Feb10).

### 2.6. Purification of IgG from Rabbit Serum

To obtain purified polyclonal immunoglobulin, whole serum supernatant was used in Montage Antibody Purification Kits with PROSEP-A (LSK2 ABA 20, Millipore). Immunized rabbit antiserum containing IgG was purified according to the manufacturer's instructions.

### 2.7. OMPs Separation by Triton X-114

To validate whether proteins are present in the leptospiral OMPs, Triton X-114 cellular fractionation was carried out in leptospiral lysates according to previously described method [14, 18]. Briefly, leptospires were grown to mid-log phase and the cells washed with phosphate buffer saline (PBS) containing 5 mM MgCl_2_ buffer and then resuspended in lysis buffer (1% Triton X-114 containing 150 mM NaCl, 20 mM Tris (pH 8), 2 mM EDTA, and 1 mM phenylmethylsulfonyl fluoride) at 4°C. Insoluble materials of leptospiral OMP were removed by centrifuging at 17,000 g for 10 min. 20 mM CaCl_2_ was added to half of the supernatant; the supernatant was warmed to 37°C and subjected to centrifugation for 10 min at 2,000 g for phase separation. Acetone precipitation was performed to separate detergent (outer membrane proteins) and aqueous (periplasmic) phases [19].

### 2.8. Rabbit Antiserum

Rabbit antiserum against* Leptospira *spp. serovar Australis (strain Ballico), Bataviae (strain Swart), Cynopteri (strain 3522C), Canicola (strain Hond Utrecht IV), Grippotyphosa (strain Moskva V), Hebdomadis (strain Hebdomadis), Javanica (Valrat Batavia 46), Pomona (strain Pomona), Icterohaemorrhagiae (strain RGA), Tarasovi (strain Perepelistin), Cellodoni (strain Cellodoni), Pyrogenes (strain Salinum), Hardjobovis (strain Sponselee), and Hardjo (strain Hardjoprajito) were obtained from WHO Collaborating Centre, Brisbane, Queensland, Australia.

### 2.9. Human Patient Samples

Five leptospirosis confirmed patient serum samples (positive control) and nonleptospirosis serum sample, which is similar like leptospirosis clinical symptoms (negative control), was obtained anonymously from the Institute for Medical Research (IMR) (http://www.imr.gov.my), Kuala Lumpur, Malaysia, for research purpose and it was incorporated into our research project. The negative control serum sample mentioned above was from a nonleptospirosis patent with clinically similar symptoms of leptospirosis. The serum samples were then evaluated by immunoblotting against rLipL21 antigens.

### 2.10. SDS-PAGE and Immunoblotting

Leptospires membrane fractions or purified proteins of rLipL21 were resolved on SDS-PAGE. The separated proteins were electrotransferred onto a nitrocellulose (NC) membrane (Millipore). The membrane was blocked with TBS (150 mM NaCl, 50 mM Tris-HCl, pH 7.4) containing 0.05% Tween 20 (T-TBS) and 3% bovine serum albumin at 37°C for 2 hrs. After the T-TBS wash, the membrane was incubated with primary antibody overnight and then washed with T-TBS. The membrane was then incubated with alkaline phosphatase conjugated secondary antibody at 37°C for 2 hrs. The NC membrane was then developed by BCIP and NBT substrates. The LipL21 band was visualized by the dark blue colour. The enzyme reaction on the membrane was terminated by washing with distilled water [20].

In order to detect recombinant protein of rLipL21, the protein was resolved on SDS-PAGE gel. The protein was then transferred to NC membranes (Millipore) and immunoblotted using the 1 : 20,000 dilution of primary rabbit antiserum (against* Leptospira *spp.) and 1 : 100 dilution of human serum (Calbiochem, Germany) overnight, followed by T-TBS wash thrice. The blot was incubated with 1 : 10,000 diluted alkaline phosphatase conjugated secondary goat anti-rabbit IgG (Calbiochem, Germany) or 1 : 10,000 diluted alkaline phosphatase conjugated secondary goat anti-human immunoglobulin (Calbiochem, Germany) at 37°C for 2 hours. The NC membrane (blot) was developed using BCIP and NBT substrates (Novagen, USA).

In order to determine if IgG created against the purified recombinant forms of these proteins could effectively bind to their cognate proteins found in the leptospires, OMP fraction and other fractions were resolved using SDS-PAGE and transferred to NC membranes. These membranes were then probed using purified anti-rLipL21-IgG at a 1 : 30,000 dilution of primary rabbit serum (anti-rLipL21) and goat anti-rabbit IgG-alkaline phosphatase (Calbiochem, Germany) at a 1 : 10,000 dilution as a secondary antibody, with BCIP and NBT substrates (Novagen, USA).

## 3. Results

### 3.1. Amplification of* lipL21*

**Figure 1:**
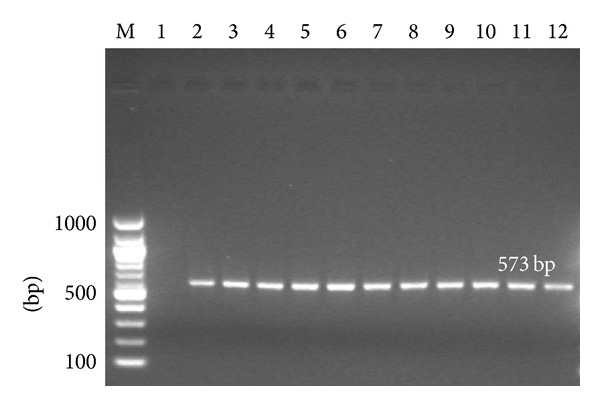
PCR amplified* lipL21* gene from different serovars: Lane M: 1 kb marker; Lane 1: nonpathogenic species* Leptospira biflexa* (strain Patoc 1); Lane 2: Canicola (strain Hond Utrecht IV); Lane 3: Hardjobovis (Sponselee); Lane 4: Autumnalis (strain Akiyami A); Lane 5: Icterohaemorrhagiae (strain RGA); Lane 6: Australis (strain Ballico); Lane 7: Pomona (strain Pomona); Lane 8: Grippotyphosa (strain Moskva V); Lane 9:* L. kmetyi *serovar Malaysia strain Bejo-iso 9^*T*^; Lane 10: Balum (strain Mus 127); Lane 11: Grippotyphosa (strain Moskva V); Lane 12: Hebdomadis (strain Hebdomadis).

Polymerase chain reaction was performed using* lipL21* gene specific primers for detection of* Leptospira* spp. The expected size of amplified* lipL21* gene from pathogenic leptospires strains was 573 bp. No amplification was observed from nonpathogenic leptospires (Figure 1) and other bacterial strains (data not shown). PCR amplification showed that* lipL21* gene is conserved among* Leptospira *spp. only, and designed primer specifically amplified the* lipL21* gene from pathogenic* Leptospira* spp. The DNA sequence was identified, analyzed, and deposited in the GenBank database (Table 2). Blast analysis showed that this sequence is 95% and 96% similar at the nucleotide and predicted amino acid levels, respectively, to the sequence of annotated gene coding for* lipL21* in the complete genome sequence of* L. interrogans* strain Lai.

### 3.2. Expression and Purification of His-Tagged LipL21 Protein

**Figure 2:**
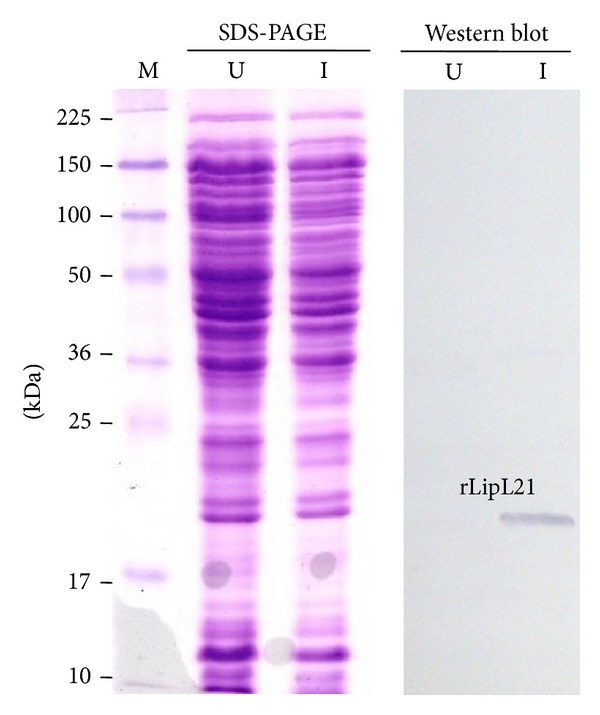
Expression analysis of rLipL21 on SDS-PAGE and Western blot. Lane U: uninduced lysates containing the pEL21 plasmid only; Lane I: auto induced lysates containing the pEL21 plasmid; Lane M: protein ladder.

The rLip21 gene was cloned in an expression plasmid with an N-terminal His tag and the construct was then transformed into* E. coli* BL21. His tag was used for the purification of recombinant LipL21 (rLipL21) by Ni-NTA affinity column. Aliquots of* E. coli*-induced cultures were analyzed on 12% SDS-PAGE and expression of rLipL21 protein was confirmed by Western blot with anti-His MAb (Figure 2). The blotted membrane indicated the presence of corresponding band in the expressed rLipL21 protein.

### 3.3. Immunoreactivity of rLipL21 Protein

**Figure 3:**
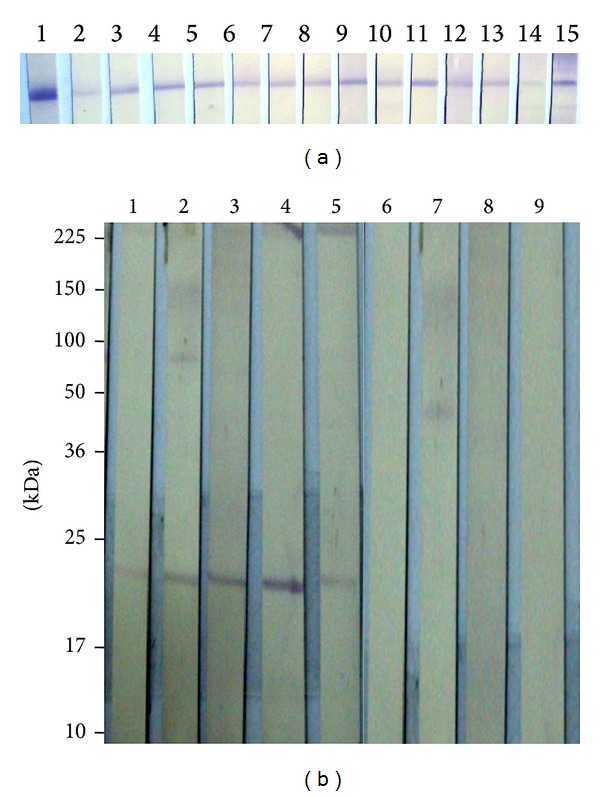
(a) Immunoblot reaction of rLipL21 protein recognized by different* Leptospira *spp. in serovar specific hyperimmune sera. Lane 1: His Tag monoclonal antibody; Lane 2: Australis (strain Ballico); Lane 3: Bataviae (strain Swart); Lane 4: Cynopteri (strain 3522C); Lane 5: Canicola (strain Hond Utrecht IV); Lane 6: Grippotyphosa (strain Moskva V); Lane 7: Hebdomadis (strain Hebdomadis); Lane 8: Javanica (Valrat Batavia 46); Lane 9: Pomona (strain Pomona); Lane 10: Icterohaemorrhagiae (strain RGA); Lane 11: Tarasovi (strain Perepelistin); Lane 12: Cellodoni (strain Cellodoni); Lane 13: Pyrogenes (strain Salinum); Lane 14: Hardjobovis (strain Sponselee); Lane 15: Hardjo (strain Hardjoprajito). (b) Immunoblots examining the reactivity of leptospirosis positive human sera with rLipL21. Lane 1–8: 0.5 *μ*g of purified recombinant rLipL21 was probed with sera from Malaysian leptospirosis patients; Lane 1–5: leptospirosis patient sera detected rLipL21; and Lane 6–9: nonleptospirosis sera (leptospiral like clinical symptoms) not detected rLipL21. Molecular weight standards are indicated in kilodaltons.

The immunoreactivity of the rLipL21 protein against human sera confirmed leptospirosis infection was assessed by immunoblot analysis (Figure 3(b)) and five sera samples from clinically confirmed leptospirosis patients (Figure 3(b)). Figure 3(b) presents the typical reaction of serum samples obtained from patients with (lanes 1 to 5) and without (lanes 6 to 9) leptospiral infection to detect the rLipL21. The rLipL21 protein with rabbit hyperimmune sera was tested. Antibody against the whole* Leptospira* spp. was detected by immunoblotting in all hyperimmune sera tested, with strong signal intensity as observed (Figure 3(a)).

### 3.4. Purification of Rabbit Anti-rLipL21 Immunoglobulins

**Figure 4:**
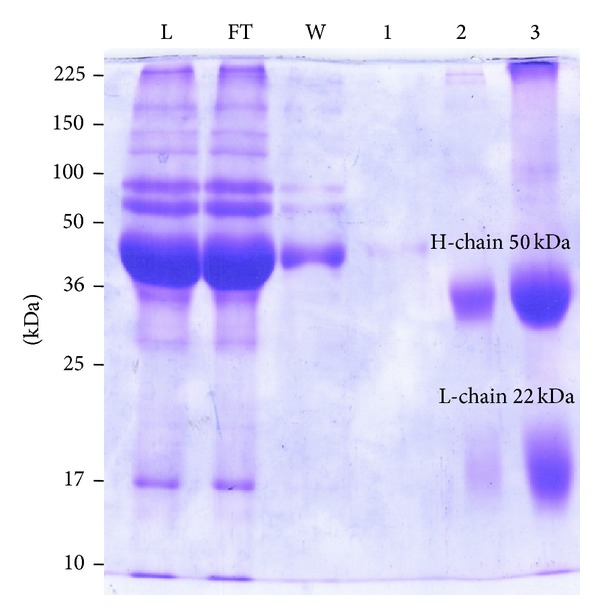
Purification of (anti-rLipL21) IgG antibody was purified from rabbit antiserum against rLipL21. Lane L: anti-rLipL21 serum; Lane FT: flow through of unbound proteins from column; Lane W: wash of nonspecific proteins from column; Lane 1: eluted fraction without protein; Lane 2: purified anti-rLipL21-IgG; Lane 3: concentrated anti-rLipL21-IgG. Molecular weight standards are indicated in kilodaltons.

SDS-PAGE analysis of the purified IgG fraction of the anti-rLipL21 antiserum revealed two bands corresponding to the heavy and light chains of IgG (Figure 4). The protein content of this fraction was 45 mg which was about one-third of primary protein content.

### 3.5. Specificity of Rabbit Anti-rLipL21-IgG Antibody

**Figure 5:**
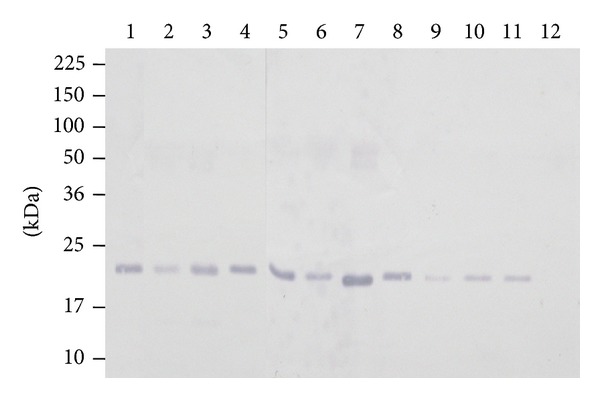
Immunoblot of panel of* Leptospira *spp. obtained by using rabbit anti-rLipL21-IgG antibody to detect single band in leptospiral LipL21 antigen in detergent phase. Lane 1: Canicola (strain Hond Utrecht IV); Lane 2: Hardjobovis (Sponselee); Lane 3: Autumnalis (strain Akiyami A); Lane 4: Icterohaemorrhagiae (strain RGA); Lane 5: Australis (strain Ballico); Lane 6: Pomona (strain Pomona); Lane 7: Grippotyphosa (strain Moskva V); Lane 8:* L. kmetyi *serovar Malaysia strain Bejo-iso 9^*T*^; Lane 9: Balum (strain Mus 127); Lane 10: Grippotyphosa (strain Moskva V); Lane 11: Hebdomadis (strain Hebdomadis); Lane 12: nonpathogenic species* L. biflexa* (strain Patoc 1). Molecular weight standards are indicated in kilodaltons.

IgG-enriched fractions (anti-rLipL21-IgG) were able to recognize leptospiral native LipL21 antigen by immunoblot, which demonstrated that the antibody was specific against LipL21 from pathogenic* Leptospira *spp. (Figure 5). Preimmune serum did not react with the rLipL21 proteins (data not shown). When these antibodies were tested against triton X-114 fraction of pathogenic serovars of* Leptospira *spp., they bound to proteins that corresponded to the molecular weight of the native protein. From this result, it can be assumed that the recombinant forms of the candidate antigens effectively mimic the properties of the native form.

## 4. Discussion

Leptospirosis is an important public health disease in endemic areas all over the world. In Malaysia, continuous presence of multiple leptospires serovars is observed in competent reservoirs like rat and other animals, and a large population of susceptible hosts [4]. Humans and animals infected with leptospires mount strong and rapid antibody responses directed against the outer surface protein, LipL21. The presence of LipL21-directed antibodies in patient serum is a reliable marker of leptospiral infection. Lipoproteins are an important antigen and play a key role in the pathogenesis of leptospirosis [14] and surface-exposed putative lipoproteins [21, 22]. Several lipoproteins and surface-exposed putative lipoproteins have been identified in* Leptospira *spp., which are associated with the outer membrane particularly those exposed on the cell surface where bacterial pathogens interact with the host [23]. These lipoproteins are maintaining the bacterial cell structure, attachment to various substrates, and immunogenicity [5]. Among these lipoproteins, the LipL21 protein has been well characterized in* L. interrogans serovar *Lai [24].* LipL21* has been reported to be conserved in pathogenic leptospiral strains and this has led to its use in PCR-based identification of* Leptospira *spp. [7]. In this study, a PCR assay was performed using* lipL21* gene specific primers for detection of* Leptospira *spp. The expected size (573 bp) of amplified* lipL21* gene was seen in 11 pathogenic* Leptospira* strains but not observed in the nonpathogenic leptospiral strain (Figure 1). Similarly, the* lipL21* gene specific primer did not amplify any other 10 bacterial strains (data not shown). The* lipL21* gene specific primer would be useful for identification of pathogenic* Leptospira* spp. These PCR results corroborated well with previous reports that LipL21 is present only in pathogenic and absent in nonpathogenic strains [12, 25].

Genomic analysis revealed that the identified LipL21 proteins of* L. interrogans* strain Pomona were found to be 97–100% amino acids similar to other pathogenic* Leptospira* spp. An alignment of the LipL21 sequence from pathogenic* Leptospira* revealed 96% to 100% identity. They also did not have any significant similarity with the proteins of other organisms [26, 27]. However, insights from recent genome projects have shown that nonpathogenic strain of* L. biflexa* LipL21 protein is an ortholog with ~50% identity present in pathogenic leptospires [26]. Its exclusive presence in pathogenic leptospires indicated that this protein could be a good candidate in developing diagnostic kits [13].

Generally, important diagnostic and vaccine markers primarily induce antibodies against surface structures. Molecular analysis of leptospiral OMP antigens is important in understanding the antibody response of hosts, because the antigenic diversity may influence the specificity and sensitivity of the serological assay. The conserved nature and high level of expression of LipL21 among pathogenic* Leptospira *spp. [7] suggest that rLipL21 immunoblot may show similar performance regardless of the local isolates.* E. coli* based expression system is now routinely used for the synthesis of recombinant proteins for a variety of purposes ranging from structural studies to the development of vaccines [11, 12, 19]. Similarly, the purification of recombinant protein is important to develop a detection system for infectious diseases. The present work adopted similar approach for expression of LipL21 protein and purification of rLipL21 in* E. coli* cells (Figure 2).

Normally,* E. coli* based IPTG/NaCl induced expression system was used for the synthesis of recombinant lipoproteins from* Leptospira *spp. [28–30]. Drawback for the use of this system includes the requirement of expression conditions such as need to follow growth of bacterial culture and add IPTG/NaCl at the proper time. An advantage of the use of Overnight Auto Induction System 1 in the present study did not require any inducer including IPTG/NaCl.

In this study, an immunoblot using the rLipL21 protein as an antigen was evaluated for the diagnosis of leptospirosis in humans. In this study, we evaluated the clinical utility and the corresponding sensitivity and specificity of recombinant LipL21 based immunoblot for the serodiagnosis of leptospirosis. The specificity of the antibody test (immunoblot) with a rLipL21 protein detected by clinically confirmed leptospirosis patient sera (Figure 3(b)). In order to confirm the similarity of the antigenic structure of the recombinant forms of the LipL21 protein, as well as to verify the cell wall localization of LipL21, we carried out a simple immunoblot study in which we tested the ability of the recombinant proteins to react with serum from a rabbit (Figure 3(b)). The strong reactivity of the recombinant forms of LipL21 with the patient serum confirms the information from studies carried out previously [12, 31]. Recombinant LipL21 was also reactive with this serum, meaning that LipL21 is immunogenic during leptospiral infection. This is probably because LipL21 protein is unique and highly conserved to pathogenic* Leptospira *spp. [12]. The specificity of LipL21 has been demonstrated to be strong immunogenic and could be an efficient tracer to LipL21 identification from* Leptospira* spp. Thus, use of the rLipL21 antigen in immunoblot has the potential to become a useful tool for serodiagnosis of leptospiral infection.

Lipoproteins may be used as novel targets for the development of infection markers and leptospirosis vaccines [5]. In pathogenic* Leptospira *spp., LipL21 has been demonstrated to be a strong immunogen, which had been considered for vaccine development [12, 27, 29]. Together, these findings suggest that rLipL21 antigen is specific and sensitive for the detection of antibodies against leptospiral infection. Both rabbit and human anti-leptospiral antibodies were found to be strongly reactive with rLipL21. Additional immunoblot results showed that the rLipL21 had the advantage of high specificity and sensitivity to leptospirosis. The present study follows the strategy of previous studies on rLipL32, rOmpL1, rLipL41, and rLigA as target antibodies used for clinical diagnosis of leptospirosis [32–35].

Although many of the cellular proteins were insoluble in the Triton X-114 or remained present in the aqueous phase, a limited number of proteins were present in the detergent phase, as previously described by Cullen et al. [12] and Haake et al. [28]. However, LipL21 protein was observed in the insoluble detergent phase and not observed in soluble phase [12]. Regardless of this, when we performed the converse study, the detergent fraction containing OMP antigen from* Leptospira* spp. and sera of immunized rabbit reacted against LipL21 antigen. Immunoblot of OMP fraction of* Leptospira* spp. using anti-rLipL21-IgG antibody revealed specificity of LipL21 antigen from pathogenic strains of leptospires (Figure 5). OMP fractions from the* Leptospira *spp. were LipL21 bound strongly detected by their anti-rLipL21-IgG antibodies. This result revealed that LipL21 is localized on the outer portion of the leptospires. This is the first demonstration of experimental characterization of the LipL21 from* L. interrogans* strain Pomona in Malaysia. Results from this study suggested that rLipL21 is a potential candidate for the serodiagnosis of leptospirosis. Further research is required to evaluate the effectiveness of this antigen with a larger number of samples obtained during outbreaks of leptospirosis. The polyclonal serum against rLipL21 protein was more specific for differentiating the pathogenic organism from nonpathogenic leptospires. Apart from these, our work revealed that the use of anti-rLipL21-IgG antibodies increases the specificity of the antigen detection. From these results, we conclude that the use of purified recombinant protein based antibody production is an appropriate and applicable method for detection of acute leptospirosis.

The polyclonal antibodies were highly sensitive but less specific in comparison to monoclonal antibodies. Monoclonal antibodies on the other hand were highly specific but less sensitive [36]. These data could have useful application to detecting LipL21 antigen from leptospires infection.

## Figures and Tables

**Table 1:** Leptospiral strains used in the LipL21 studies.

Leptospira	Species	Serovar	Strain
Pathogenic	*L. interrogans *	Pomona	Pomona
	*L. interrogans *	Canicola	Hond Utrecht IV
	*L. interrogans *	Hardjo	Sponselee
	*L. interrogans *	Autumnalis	Akiyami A
	*L. interrogans *	Djasiman	Djasiman
	*L. interrogans *	Grippotyphosa	Moskva V
	*L. interrogans *	Hebdomadis	Hebdomadis
	*L. interrogans *	Javanica	Valdrat Batavia 46
	*L. interrogans *	Australis	Ballico
	*L. icterohaemorrhagiae *	Icterohaemorrhagiae	RGA
	*L. kmetyi *	Malaysia	Bejo-Iso 9^*T*^

Nonpathogenic	*L. biflexa *	Patoc	Patoc 1

**Table 2:** *LipL21* gene accession number in the GenBank.

Pathogenic strains	GenBank
*L. interrogans* strain Pomona	EU244328
*L. interrogans* strain Ballico	FJ853169
*L. interrogans* strain Hond Utrecht IV	FJ853170
*L. interrogans* strain Akiyami A	FJ853171
*L. interrogans* strain Hebdomadis	FJ853172
*L. interrogans* strain RGA	FJ853173
*L. interrogans* strain Moskva V	FJ853174
*L. interrogans* strain Djasiman	FJ853175
